# Shortening of 3′UTRs Correlates with Poor Prognosis in Breast and Lung Cancer

**DOI:** 10.1371/journal.pone.0031129

**Published:** 2012-02-08

**Authors:** Antonio Lembo, Ferdinando Di Cunto, Paolo Provero

**Affiliations:** Department of Genetics, Biology and Biochemistry and Molecular Biotechnology Center, University of Turin, Turin, Italy; Sun Yat-sen University Medical School, China

## Abstract

A major part of the post-transcriptional regulation of gene expression is affected by trans-acting elements, such as microRNAs, binding the 3′ untraslated region (UTR) of their target mRNAs. Proliferating cells partly escape this type of negative regulation by expressing shorter 3′ UTRs, depleted of microRNA binding sites, compared to non-proliferating cells. Using large-scale gene expression datasets, we show that a similar phenomenon takes place in breast and lung cancer: tumors expressing shorter 3′ UTRs tend to be more aggressive and to result in shorter patient survival. Moreover, we show that a gene expression signature based only on the expression ratio of alternative 3′ UTRs is a strong predictor of survival in both tumors. Genes undergoing 3′UTR shortening in aggressive tumors of the two tissues significantly overlap, and several of them are known to be involved in tumor progression. However the pattern of 3′ UTR shortening in aggressive tumors *in vivo* is clearly distinct from analogous patterns involved in proliferation and transformation.

## Introduction

MicroRNAs and other trans-acting factors that bind mRNA regulate protein levels by preventing mRNA translation and inducing its degradation. The key role played by this mechanism of post-transcriptional regulation in development [1] and disease [2] is emerging from an ever growing body of experimental results.

Many individual microRNAs have been shown to be aberrantly expressed in several types of cancer [2]. While some microRNAs act as oncogenes, in most reported cases it is the loss of microRNA expression that correlates with the onset or the aggressiveness of the tumor. A picture emerges in which microRNAs keep cells from inappropriately proliferating and invading the surrounding tissues [3], [4] by repressing the expression of their targets.

In a heterogeneous cell population, selection will favor the cells that are able to escape such control. This can be done either by suppressing the expression of the individual microRNAs or, perhaps more efficiently, by interfering globally with the mechanism of regulation by microRNAs, for example by decreasing expression of genes involved in the microRNA pathway such as *DICER1* [5], [6] or *TARBP2* [7].

Another way of escaping microRNA control is to remove their binding sites by expressing shorter transcripts: often this can be achieved without altering the gene product since the microRNA binding sites are mostly located within 3′ untranslated regions. Many mRNAs contain 3′ UTRs with alternative polyadenylation (APA) sites that can be used to produce transcripts of different length (see e.g. the review [8]).

Indeed it was shown that proliferating cells [9], cancer cells [10], [11], early-stage embryonic cells [12], [13] and induced pluripotent stem cells [14] express shortened 3′ UTRs with fewer microRNA binding sites. This way of escaping microRNA control by removing binding sites in *cis* has the advantage of being more fine-tunable than the removal in *trans* of the microRNA, as specific mRNA targets can be selectively released from regulation.

We therefore asked whether the same phenomenon exists in tumors, and in particular whether shorter 3′ UTRs correlate with tumor aggressiveness and shorter survival times. Such correlation was previously shown in a mouse model of lymphoma in [15]. To address this question we took advantage of the extensive cancer gene expression datasets available in the public domain produced with Affymetrix chips of the U133 generation, and including patient survival and other clinical data.

While these chips were not originally designed for discriminating between alternative transcripts, they can be used [12] to answer our question, at least partially. Indeed, on one hand, most probes match the 3′ region of the target transcripts and, on the other, probes can be reorganized into user-defined probesets. Exon arrays would be the obvious choice, but the number of large-scale cancer datasets obtained with this platform is still limited.

We thus defined custom probesets matching 3′ UTR regions separated by APA sites: this turned out to be possible for thousands of transcripts, for which we determined the relative expression of the long and short forms of the 3′ UTR in each patient. The correlation between these data and patient survival could then be studied to determine whether the expression of longer or shorter 3′ UTRs correlates with cancer aggressiveness.

## Results

Affymetrix chips of the HG-U133 series have been extensively used in studies of gene expression in cancer. Several datasets are available containing hundreds of patients together with survival data and other clinical information. Most probes are located at the 3′ end of the target transcript. Even though the single probes were organized by the manufacturer in standard probesets, they can also be reorganized into custom probesets [16], either to take advantage of improvements in the annotation of the genome or to perform special-purpose analyses. These features of the Affymetrix chips were previously exploited in [12], [14] to study 3′ UTR shortening in mouse development and in induced pluripotent stem cells.

We created a custom Chip Description File (CDF), *i.e.* a custom set of probesets, for the HG-U133A, HG-U133B and HG-U133_Plus_2 chips, matching alternative transcripts that differ in the portion of the 3′UTR they express. Alternative transcripts of this type are generated using APA sites, whose location can be predicted by sequence analysis and comparison with cDNA/EST sequences. We used the polyA_DB database [17], [18] to obtain a collection of predicted APA sites.

Given a transcript with an APA site, we divided the Affymetrix probes matching the transcript into two sets, 5′ and 3′ probes, relative to the APA site. We then created separate probesets from the 5′ and 3′ probes. The software package we used [16] recommends creating probesets containing at least three probes. With this criterion, we could build separate 5′ and 3′ probesets for 2,706 transcripts represented in the HG-U133A and HG-U133B chips. For 685 transcripts it was possible to create 5′ and 3′ probes for more than one APA sites.

The long version of a transcript will hybridze with both the 5′ and 3′ probes, while the short version generated by the APA site will hybridize only with the 5′ probes. Denoting with 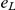 (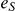) the expression level of the long (short) form, the signal from the 5′ probeset will be
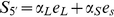
(1)where 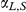 represent the affinities of the long and short forms for the 5′ probeset. Similarly the signal from the 3′ probeset will be
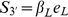
(2)so that the Expression Ratio Index (ERI) defined as their ratio
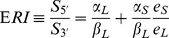
(3)is a linear function of the expression ratio between the two alternative transcripts, and can be used to study their relative prevalence. On the other hand an overall change of expression of the transcript, which does not alter the relative amounts of the two forms, does not change the ERI value.

We can thus compare the ERI values in two biological samples to determine whether the long and short forms of the transcript are expressed in different ratios. A higher ERI correponds to a higher prevalence of the short form.

### Comparison with PCR-based results

We validated the use of the ERI to infer relative expression of long and short 3′ UTR forms by comparing the PCR-based data reported in Ref. [10] in various cancer cell lines to microarray data obtained on HG-U133_Plus_2 array on some of the same cell lines and available from GEO repository accession GSE10843.

**Figure 1:**
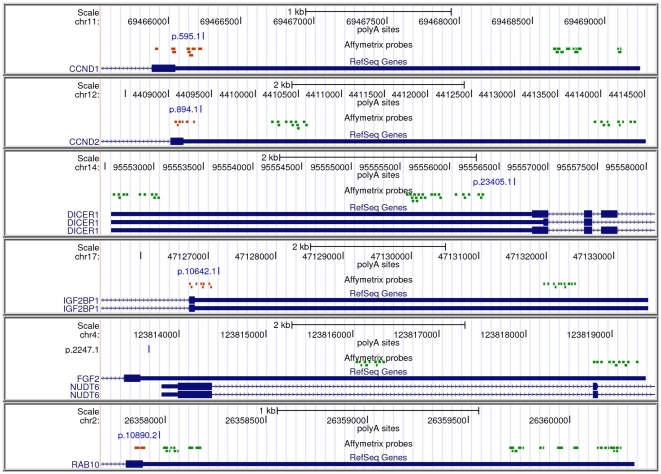
Affymetrix probes and APA sites in the 3′ UTR of six genes studied in Ref. [**10**]. The probes shown in red are used, possibly together with probes located in the coding region, to build the 5′ probeset. The probes shown in green are used to build the 3′ probeset. For DICER1 and FGF2 it is not possible to study the APA site since all probes lie beyond it.

For 4 of the 6 genes shown in Figure 2 of [10] we could build separate probesets for the regions upstream and downstream of the relevant APA site (*CCND1*, *CCND2*, *IGF2BP1*, *RAB10*). For *FGF2* and *DICER1* this was not possible, because all Affymetrix probes lie distal to the APA site. Incidentally, this shows that expression data for these genes obtained with HG-U133 platforms must be interpreted with great caution, because the microarray cannot hybridize to the transcript expressing the shorter 3′ UTR form, which might very well be the one associated with higher protein expression. The relevant probes are shown in Figure 1.

As explained above, we expect the ERI to be a linear function of the expression ratio of short vs. long 3′UTR. Therefore we expect the ERI as computed from microarray data to correlate positively with direct measurements of expression ratios (in the absence of all experimental variability and noise we would expect a Pearson correlation coefficient equal to 1). The authors of [10] report in their Figure 2 the expression ratios of short vs. long 3′UTR for several cell lines originating from breast, colon and lung tumors. Each ratio is normalized to the same ratio in the corresponding normal tissue: therefore we computed, separately for each tissue of origin and each of the four genes, the Pearson correlation coefficient between the ERI derived from the micorarray data and the normalized ratio reported by [10], computing a total of 6 correlation coefficients. All of them turned out to be positive (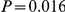, binomial test), varying between 0.34 for *CCND1* in lung cancer cells and 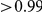 for *CCND1* in breast cancer cells. These results convinced us that the ERI we defined can indeed be used to probe the differential expression of alternative 3′ UTR forms.

**Figure 2:**
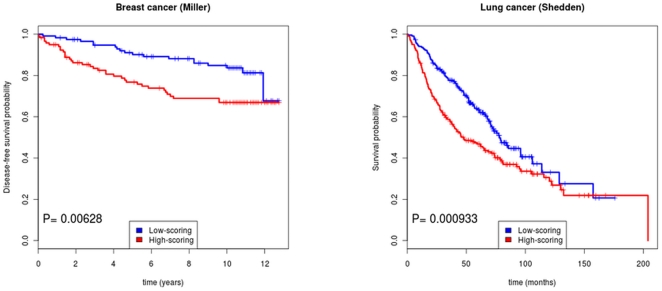
Predictive power of the prognostic score based on ERI in two dataset not used to derive the signature. (A) the Miller breast cancer dataset [20] and (B) the Shedden lung cancer dataset [32]. Patients are divided into groups using the median score as a cutoff.

### Comparison with high-throughput sequencing results

To validate the method also at a more global scale we compared its results to those obtained on breast cancer cell lines using high-throughput sequencing in a recent publication [11]. Here the authors studied differential APA usage between two breast cancer cell lines (MDA-MB-231 and MCF7) and a cultured mammary epithelial cell line (MCF10A).

We used microarray data for the three cell lines available from the GEO database under accession GSE10890, and selected untreated samples of the three cell lines of interest (7 samples for each cancer cell line and 2 samples of MCF10A). The platform used here is the HG-U133_Plus_2, for which we could build separate 5′ and 3′ probesets for 6,045 APA sites in 3,542 unique genes. For each APA site and each pair of cell lines we identified differential APA usage with a 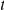-test on the ERI values followed by multiple testing correction with the Benjamini-Hochberg procedure and a False Discovery Rate (FDR) cutoff of 5%.

We thus obtained lists of genes with significantly shortened or lengthened 3′ UTR for the three possible comparison, thus a total of 6 lists, to be compared with the analogous lists compiled from high-throughput sequencing in [11]. The overlap turned out to be statistically significant (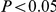) in 5 out of 6 cases, the most significant one corresponding to 3′ UTRs that are shortened in MDA-MB-231 cells compared to MCF7 cells (318 genes concordantly detected by microarray and sequencing, compared to 227 expected by chance, 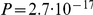, exact Fisher test). The only case in which the overlap was not statistically significant was 3′ UTRs that are lengthened in MCF7 cells compared to MCF10A, which is also the least common case according to both approaches. We concluded that our method, when used on a genome-wide scale, gives results that are in good agreement with unbiased approaches such as high-throughput sequencing.

### Aggressive breast cancers express transcripts with shorter 3′ UTRs

Using the strategy described above, we evaluated the relative expression of long and short 3′ UTR forms in a large, population-derived dataset of 159 cases of breast cancer in which gene expression profiles were measured using both HG-U133A and HG-U133B platforms [19], which we will refer to as the Pawitan dataset.

We divided the patients into two groups based on outcome (relapse) at 5 years. The large sample size allowed us to use a non-paramteric test, so we replaced the 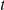-test used for cell lines with a Mann-Whitney 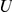 test to find the transcripts for which the ERI is significantly different between the two groups, *i.e.* the transcripts for which the relative prevalence of long and short forms of the 3′ UTR differs between the two groups of patients. After correcting for multiple testing using the Benjamini-Hochberg procedure we found, at 5% FDR, 35 APA sites in 28 different transcripts for which the relative expression of the short and the long form is significantly different between the two outcome groups (Table S1). Of these, 34 showed higher short/long expression ratio in the poor-outcome group and only one showed the opposite behavior: we conclude that, globally, the expression of shorter 3′ UTRs correlates with poor outcome, in qualitative agreement with results obtained from cell lines [10].

### A prognostic signature based on APA site usage

These results suggest that the relative prevalence of the short and long forms of the 35 APA sites can be used to stratify patients into risk classes. We thus developed a prognostic score based on the ERI of these APA sites. The prognostic score of a patient was defined as a linear combination of the ERI values of the 35 sites in the signature. The coefficients in the linear combination are determined from Cox univariate analysis as detailed in the Methods. Patients with high prognostic scores are characterized by relatively higher expression of the isoform associated to poor outcome (the short form for all but one APA site).

The prognostic score can be used to separate patients in two groups based on a cutoff. When applied to the Pawitan dataset, using the median score as a cutoff, the two groups of patients are significantly different in survival probability (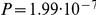, log-rank test). This is however expected, since the APA sites in the signature were chosen based on their differential ERI in the same dataset.

We thus used an independent, population-derived breast cancer dataset of 236 samples measured with the same platforms [20], the Miller dataset, to verify the robustness of the prognostic signature. The prognostic score of each patient of this dataset was computed as in the case of the Pawitan dataset, and the median score was used to divide the patients into two groups. Importantly, the coefficients used were the ones derived from the Pawitan dataset, so that the Miller dataset was not used at all in determining the signature. The two groups of patients differed significantly in disease-specific survival probability (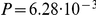, Figure 2A). Therefore the short/long expression ratio of the 35 APA sites identified in the Pawitan dataset can be used to create a robust outcome predictor based exclusively on the relative prevalence of alternative 3′ UTRs.

### Differential synthesis vs. differential degradation

**Figure 3:**
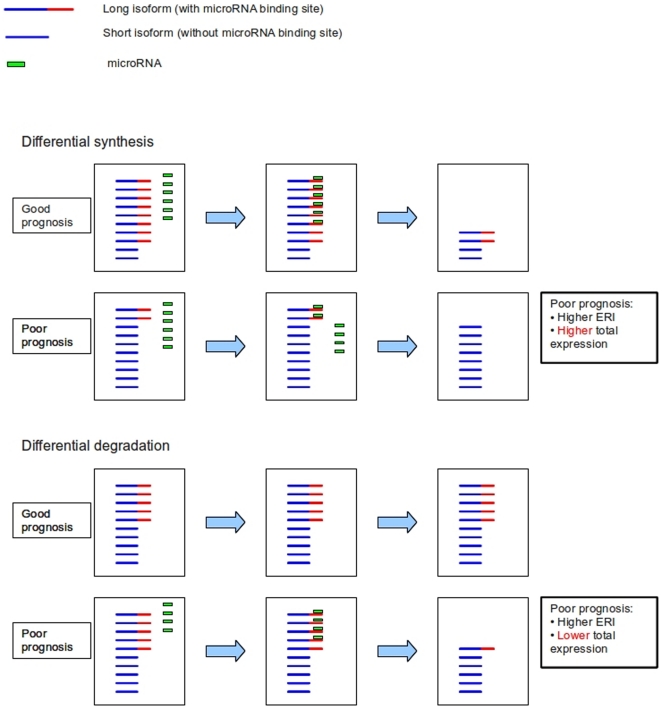
Schematic representation of two possible mechanisms leading to higher ERI in poor-prognosis tumors. (A) Differential synthesis: the shorter isoform is synthesized in a higher proportion in the poor-prognosis tumors, leading to less degradation by microRNAs. (B) Differential degradation: the isoforms are produced in the same proportion in the two cases, but a microRNA expressed exclusively in the poor-prognosis tumors selectively degrades the long isoform. In both cases we expect a higher ERI (relative prevalence of short form) in the poor-prognosis group. However in case (A) the overall expression of the two isoforms is expected to be higher in the poor-prognosis case, while the opposite is expected in case (B).

The observed higher ERI in poor-outcome tumors could be due either to altered ratios in the synthesis of the two isoforms, or to altered degradation rates. As an example of the latter mechanism, assume a microRNA, able to bind the long isoform of a transcript but not the short one, is expressed at higher levels in poor-outcome tumors. Then even if the two isoforms are synthesized at the same rate, the long one will be degraded faster in the poor-outcome tumors, leading to higher ERI. At least in the simplest case in which only one of the two mechanisms is at work it is possible to distinguish between them by looking at the differences in the total expression of the transcript between poor- and good-outcome cases (Figure 3).

First suppose differential synthesis is involved, *i.e.* relatively more of the short form is synthesized in poor-outcome tumors. Then the decay rate in this class of tumors will be lower, because of the loss of microRNA binding sites: hence we expect a higher total expression of the transcript in poor-prognosis tumors.

On the contrary, if the ratio of synthesized isoforms is the same in the two classes of tumors, but microRNAs able to degrade the transcript are more abundant in the poor-outcome cases, we expect higher degradation rates in the latter class, and thus lower total expression of the the transcript in poor-prognosis tumors.

Since the probes located 5′ of the APA site are able to hybridize to both isoforms, they can be used to evaluate the total expression of each transcript. Therefore we can distinguish between the two cases by looking at the correlation between differences in ERI and differences in signal from the 5′ probesets: if differential synthesis (degradation) is the dominant mechanism, we expect positive (negative) correlation between these two quantities.

We thus computed, for each transcript, the difference in median expression of the 5′ probeset between poor- and good-outcome tumors, and its correlation with the difference in median ERI. To avoid overcounting transcripts with more than one APA site we included only the 5′-most site for each transcript. We obtained a positive and highly significant Spearman correlation coefficient (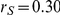, 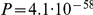). While this analysis is based on a simplified model, it suggests differential synthesis is a significant mechanism leading to 3′ UTR shortening in aggressive tumors.

### 3′ UTR portions containing microRNA binding sites are preferentially lost in aggressive tumors

If the expression of shorter 3′ UTRs confers a selective advantage by allowing escape from microRNA-mediated repression, we expect the 3′ UTR portions containing microRNA binding sites to be lost in aggressive tumors more often than those which do not contain them. To test this expectation we defined as “microRNA-relevant” the APA sites removing at least one predicted microRNA binding site from the transcript. For each APA site, we took the difference in mean ERI between poor- and good-prognosis tumors as a measure of 3′ UTR shortening: microRNA-relevant sites showed significantly more shortening than irrelevant ones (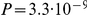, Mann-Whitney U test). Thus the shortening of 3′ UTRs in aggressive tumors is more pronounced for those transcripts for which it leads to loss of microRNA binding sites.

The same analysis can be performed for individual microRNAs, by comparing APA sites that are relevant, in the sense defined above, for specific microRNAs to irrelevant ones. At 10% FDR we found 5 microRNAs significantly associated with 3′ UTR shortening in aggressive tumors: miR-7, miR-96, miR-200bc/429, miR-299-5p and miR-496. No microRNA was significantly associated with 3′ UTR shortening in good-prognosis tumors.

Since these are the microRNAs whose regulatory activity is partly suppressed by the removal of their binding sites in shortened 3′ UTRs, we would expect the microRNA themselves to have tumor-suppressor activity. Indeed three of these microRNAs were previously shown to have such effect in breast cancer cell lines: the miR-200bc/429 family negatively regulates EGF-driven cell invasion, viability and cell cycle progression [21], and suppresses the epithelial to mesenchymal transition [22]; tumor suppressor activity of miR-7 in breast cancer cell lines was shown in [23], [24], and its role in preventing resistance to chemotherapy in [25], [26] (note, however, that miR-7 correlates with poor prognosis in a specific molecular class of breast cancer [27]); decreased expression of miR-299-5p was associated to increased tumorigenic potential [28]. Our results suggest that cancer cells can overcome regulation by these microRNAs not only by decreasing their expression, but also by preventing them from binding to their targets.

More puzzling is the appearance of miR-96, which, on the contrary, was shown to have proliferation-promoting activity by repressing FOXO transcription factors [29], [30]. However, as discussed above, the effect of removing selected targets from microRNA control can be vastly different from the effect of simply removing of repressing the microRNA itself.

### Comparison with results in cell lines and normal tissues

Since 3UTR shortening has been previously associated to proliferation, one possibility to explain the specific effects that we detected could be the increased proliferation that characterizes aggressive tumors. Therefore we asked to what extent the transcripts showing differential APA usage in poor *vs.* good-prognosis patients coincide with those found in recent genome-wide studies of APA usage in normal and cancer cell lines. However, no significant overlap was found between the 28 transcripts involved in our breast cancer signature and the 980 mouse transcripts showing differential APA usage in mouse activated lymphocytes [9].

The same lack of statistically significant overlap was found when comparing our results to the ones obtained comparing different breast cancer cell lines to a normal breast cell line in [11]. Such negative result holds also when using our own microarray-derived results for the same cell lines (from the same dataset GSE10890 used above to check our algorithm), suggesting that the lack of overlap is not due to different biases between sequencing- and microarray-based measurements.

On the contrary, a pattern of differential 3′ UTR usage similar to the one we find in aggressive vs. less aggressive breast tumors is found when comparing breast tumor samples (independently of aggressiveness) to healthy tissue: using the data of Ref. [31] (accession GSE3744 in GEO) we compared 38 sporadic tumor samples to 7 normal breast samples. We observed, as expected, a large number of APAs displaying 3′ UTR shortening in cancer vs normal tissues (1,222 APAs corresponding to 921 unique genes at 5% FDR) and only 212 APAs (189 genes) showing the opposite behavior. The platform used here is the HG-U133_Plus_2, which allows us to analyze a total of 4,933 APAs (3,452 genes). The genes undergoing 3′ UTR shortening significantly overlap with the ones found above from the Pawitan dataset (21 genes in common compared to 6.98 expected by chance, 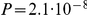).

Therefore while widespread 3′ UTR shortening is observed in many different contexts, all generically related to increased proliferation, the transcripts involved are significantly different in the different contexts, even within the same tissue of origin. In particular we observe similar patterns of 3′ UTR shortening when comparing breast tumors to normal tissues and when comparing breast tumors of different aggressiveness: however this pattern is markedly different from what observed in breast cancer cell lines, suggesting that the use of cancer cell lines in studying this particular phenomenon might not be ideal.

### 3′ UTR shortening in aggressive lung cancer

We asked whether the pattern of 3′UTR shortening found in aggressive breast tumors was specific of breast cancer or relevant to other tumors. Specifically we considered a large lung cancer dataset [32], containing expression profiling of more than 400 lung adenocarcinomas. The 35-APA signature found in breast cancer turned out to be predictive of survival also in these patients: when computing the prognostic score of each patient using the signature and dividing them into two groups using the median score as cutoff we found a significant difference in patient survival (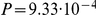, Figure 2B). This result suggests that the pattern of 3′ UTR shortening in aggressive lung cancer is similar to the one observed in breast cancer.

Therefore we repeated the analysis leading to the breast cancer signature directly on the lung cancer dataset. To account for the shorter overall survival of lung cancer patients we defined the poor-prognosis group to include all patients who died of the disease within 30 months. At 5% FDR we found a total of 319 APA sites with differential usage between good- and poor-prognosis patients (Table S2): in 258 (81%) of these the shorter isoform is predominantly expressed in poor-prognosis patients.

The overlap between the breast and lung cancer results is highly significant: 11 genes (Table 1) show significant 3′ UTR shortening in poor-prognosis patients of both cancers, compared to an overlap of 2.4 genes expected by chance (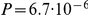). This result is surprising given the lack of significant overlap with results in cell lines discussed above, and suggests the existence of a specific program of 3′ UTR shortening associated with tumor aggressiveness in different tissues. This program is, on one hand, largely shared between breast and lung tumors, and, on the other, clearly distinct from analogous programs associated with physiological proliferation [9] and cell transformation [11].

**Table 1:** Genes showing significant 3′ UTR shortening in poor-prognosis patients of both breast and lung cancer.

Gene Symbol	Gene
*ARL1*	ADP-ribosylation factor-like 1
*AURKA*	aurora kinase A
*CACYBP*	calcyclin binding protein
*CAV2*	caveolin 2
*CENPN*	centromere protein N
*KIAA0101*	KIAA0101
*RAB27A*	RAB27A, member RAS oncogene family
*RAD51*	RAD51 homolog (RecA homolog, E. coli) (S. cerevisiae)
*SPARC*	secreted protein, acidic, cysteine-rich (osteonectin)
*TIMELESS*	timeless homolog (Drosophila)
*TIMM17A*	translocase of inner mitochondrial membrane 17 homolog A (yeast)

A first interesting observation about these genes is that many of them have been positively implicated in breast and/or lung cancer progression and resistance to therapy: *AURKA* [33], [34], *CAV2* [35], *RAB27A* [36], *RAD51* [37]–[39], *TIMELESS* [40], *TIMM17A* [41]. On the other hand there is evidence of a tumor-suppressing role of *SPARC* [42], [43].

The available information about the function of these genes suggests that one of the mechanisms by which they may promote cancer aggressiveness could be through increasing cell migration and invasiveness. Indeed, the overexpression of AURKA in breast cancer cells has been reported to increase cell migration through ADF/cofilin pathway [33]. A related action on cytoskeleton could be exerted by CACYBP [44], although the results for its implication in breast cancer are seemingly contradictory [45], [46]. Caveolins are well known modulators of cell motility and migration [47] and are significantly overexpressed in breast cancer cells characterized by basal-like and triple-negative immunophenotype [48]. RAB27A was shown to confer invasive and metastatic phenotypes on breast cancer cells by promoting the secretion of insulin-like growth factor-II [36]. Finally, SPARC influences the synthesis of extracellular matrix, elicits changes in cell shape, inhibits cell-cycle progression [49]. An even more striking observation is that three gene in the list, i.e. AURKA [50], [51], KIAA0101 [52] and RAD51 [53]–[55] are localized to centrosomes, may affect centrosome duplication and are capable to physically or functionally interact with the tumor suppressor gene BRCA1. A strong role in control of genomic stability for the genes in our signature is further indicated by the facts that CENPN is a factor required for centromere assembly [56] and that TIMELESS plays a crucial role in the ATM/ATR growth arrest response induced by DNA-damage and in the G2/M checkpoint [57].

### Extension to other datasets and tumor types

To further investigate the robustness and general applicability of the two signatures derived from breast and lung cancer datasets we applied them to several other datasets available from GEO and for which both CEL files and survival data are available. Using the breast cancer-derived signature of 35 APAs we systematically observe, with the only exception of the colon cancer dataset of Ref. [58], shorter survival for high-scoring patients, even if this difference reaches statistical significance only in some cases (Figure S1). Also in this case the signature was built using the APAs and the coefficients derived from the Pawitan breast cancer dataset. In particular for breast cancer we observe that statistical significance is reached for the datasets including lymph-node positive patients [19], [20], [59], but not for those including only lymph-node negative patients [60], [61]. This might suggest that shortening of 3′ UTRs becomes important in the advanced stages of the disease. The lung-cancer derived signature shows similar results, but appears to be significantly less robust (Figure S2).

## Discussion

We have shown that 3′UTR shortening in specific mRNAs correlates with poor prognosis in both breast and lung cancer, and that a significant part of this phenomenon is attributable to differential synthesis of alternative isoforms rather than to their differential degradation. Several genes involved in tumor progression and resistance to therapy express shortened 3′ UTRs in aggressive tumors, supporting the notion that tumor cells gain an evolutionary advantage by removing these genes from the control of trans-acting regulators acting on the 3′ UTR.

The fact that 3′ UTRs are globally shorter in aggressive tumors could be expected, since shortening of 3′ UTRs in various proliferative contexts have been reported in the literature [9]–[15]. However our analysis shows that there are specific patterns of 3′ UTR shortening for different proliferative contexts. In particular the pattern of 3′ UTR shortening associated to aggressive breast and lung cancer does not overlap significantly with what is observed in proliferating T cells [9] or even in breast cancer cell lines [11]. Since the latter negative result is obtained also when cancer cell lines are analyzed with the same microarray-based method used for cancer patients, it is unlikely to be artefactual. These results suggest that cancer cell lines might not be a suitable environment to study alternative APA usage in cancer, in agreement with the results of [11].

It is therefore all the more striking that, on the contrary, there is a significant overlap between between genes undergoing 3′ UTR shortening in aggressive breast and lung cancer. We thus suggest the existence of a specific pattern of 3′ UTR shortening associated with tumor aggressiveness, significantly shared between breast and lung cancer, but clearly distinct from other such patterns associated with proliferation and transformation.

The available information about genes that compose this shared signature is very well consistent with their causal involvement in the increased aggressiveness of tumors. Indeed, the expression variation resulting from variation of their 3′-UTR length could, on one hand, affect their invasive properties and, on the other hand, affect their propensity to undergo centrosome amplification, aberrant mitosis and resistance to DNA damage. Considering that AURKA can play a role in all these processes [33], [50], [51], our results further underscore its importance as a critical pharmacological target for preventing cancer progression.

Individual *cis*-acting genetic hits involved in 3′ UTR shortening in cancer have been documented. For example in 7 out of 15 cases of mantle cell lymphoma expressing a truncated *CCND1* mRNA [62] a genomic deletion in the 3′ UTR region was detected, and in 3 other cases a point mutation led to the creation of premature polyadenylation sites. However the effect we observed seems to involve too many genes to be explained solely or predominantly in this way. Many regulatory programs involving alternative usage of APA sites in various physiological contexts have been demonstrated, so that it appears likely that cancer cells could exploit a similar program to gain an evolutionary advantage.

Such a mechanisms would involve the binding of trans-acting factors to specific sites in the vicinity of the polyA site, as shown for example in [63] where it was shown that differential polyA usage in different human tissues correlates with overrepresentation of specific cis-elements. However a similar analysis in our case did not reveal any statistically signifcant pattern. This could be due either to a lack of statistical power (possibly related to the limited number of transcripts we can study) or to the fact that the relevant cis-elements in the case of cancer recognize structural rather than sequence features.

The most significant limit of the approach we presented here is the use of 3′-based chips. This choice was due to the large availability of gene expression profiles with survival information, but severely limits the number of genes that can be analyzed (up to 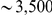 for the HG-U133_Plus_2 platform). We have shown that the alternative 3′ UTR usage of these genes can be reliably inferred from the microarray data and that it is biologically and clinically relevant; however since we cannot assume these genes to be an unbiased subset of the whole genome, all inferences about the global pattern of alternative 3′ UTR usage must be taken with great caution. Future work based on RNA-sequencing or exon arrays will be needed to study the overall usage of alternative 3′ UTRs in aggressive tumors in a less biased way.

Moreover, the analysis of datasets combining expression profiling and cancer genome sequencing data will determine what proportion of the effect is due to individual genetic hits and to changes in the regulation of mRNA polyadenylation and cleavage. Identification of possible trans-acting factors that may mediate the tumor progression-specific 3′ UTR shortening that we here described could potentially lead to novel therapeutic targets.

## Methods

### Generation of custom Chip Description Files

The sequence of all human mRNAs included in Refseq database was obtained from the UCSC genome browser. Form the same source we obtained the location of tha APA sites identified in the polyA_DB database [17], [18]. Each APA site was used to divide a mRNA sequence into two fragments (5′ and 3′ of the APA site). These fragments were then used as input for the Bioconductor package “altcdfenvs” [16], which generates the custom CDF. Affymetrix gene expression data can be re-analyzed with a custom CDF if the raw data (CEL files) are available.

### Evaluation of 3′ UTR shortening

Expression data for the 5′ and 3′ probesets included in the custom CDF were obtained using Robust Multi-Array Average (RMA) [64]. For each APA site and each biological sample, we computed the Expression Ratio Index (ERI) defined in Eq. 3 as the ratio of RMA expression values. A software package containing the custom CDF files and an R script to compute the ERIs is provided as Software S1).

A Mann-Whitney 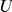 test was used to determine which APA sites showed significantly different ERI between the two group of patients (defined on the basis of survival status at 5 years in breast cancer and 2.5 years in lung cancer). Multiple testing was controlled with the Benjamini-Hochberg procedure.

### Prognostic score based on alternative 3′ UTR usage

To define a prognostic score based on ERI values we first computed, for each of the 35 APA sites differentially used in the Pawitan dataset, its Cox univariate score 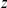. This score measures the correlation between a continuous quantity (in our case the ERI) and patient survival. A positive (negative) 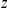 implies a negative (positive) correlation between ERI and survival.

We then defined the prognostic score of a patient as:
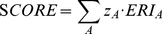
(4)where the sum is over the 35 APA sites, 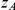 is the Cox score and 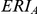 is the ERI value of the APA. High-scoring patients are thus characterized by high ERI values (*i.e.* high prevalence of the short 3′ UTR isoform) for those APA sites that are most strongly correlated to poor survival. The 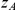 values for the 35 APA sites can be found in Table S1.

The prognostic score of each patient in each dataset was computed using Eq. 4, in which the 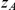 coefficients are always the ones derived from the Pawitan datasets. The predictive power of the prognostic score was evaluated in each dataset by dividing the patients into two groups using the median score as cutoff, and using a log-rank test to compare the cumulative survival probabilities of the two groups.

### Cancer datasets

The cancer datasets we used for developing and testing the prognostic signatures were all downloaded from the Gene Expression Omnibus (GEO) repository, with the exception of the lung cancer dataset of [32] which was obtained from the caArray repository (https://array.nci.nih.gov/caarray/). We used a total of 6 breast cancer datasets (GSE7390 [61], GSE20685 [65], GSE3494 [20], GSE1456 [19], GSE2990 [59], GSE2034 [60]), one colon cancer dataset (GSE14333 [58]), three lung cancer datasets (the Shedden dataset mentioned above plus GSE3141 [66] and GSE14814 [67]) and one melanoma dataset (GSE22138 [68]).

### Prevalence of microRNA binding sites in shortened 3′ UTRs

An APA site was defined to be relevant to a specific microRNA if the sequence located 3′ of the site contained one or more binding site predicted by Targetscan [69], as reported in the corresponding UCSC track. An APA site was defined to be microRNA-relevant if it was relevant to one or more specific microRNAs. The ERI values of microRNA-relevant vs. irrelevant APA sites were compared using a Mann-Whitney 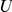 test, with multiple testing controlled with the Benjamini-Hochberg procedure.

## Supporting Information

Figure S1 Performance of the signature derived from the Pawitan breast cancer dataset as a predictor of survival in other cancer datasets. P-values are from log-rank tests.
(TIFF)

Figure S2 Performance of the signature derived from the Shedden lung cancer dataset as a predictor of survival in other cancer datasets. P-values are from log-rank tests.
(TIFF)

Table S1APA sites showing significantly different Expression Ratio Index between bad- and good-prognosis breast cancer patients (Pawitan dataset). The columns are: (1) APA site, identified as in the PolyADB track of the UCSC genome browser (human genome release hg18); (2) Entrez gene id; (3) Official gene symbol; (4) gene name; (5) difference in logarithmic ERI betwee poor- and good-prognosis patients: a positive value indicates shortening of the 3′ UTR in poor-prognosis patients; (4) P-value from Mann-Whitney U test; (5) Q-value from Benjamini-Hochberg correction; (6) 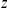-score from Cox univariate analysis.(XLS)

Table S2 APA sites showing significantly different Expression Ratio Index between bad- and good-prognosis lung cancer patients (Shedden dataset). Columns as in Supporting Table S1.
(XLS)

Software S1 An R package containing the custom CDF files and a script to compute the ERI values.
(BZ2)
