# Supplementary figures and images for: Shortening of 3′UTRs Correlates with Poor Prognosis in Breast and Lung Cancer
Source: PLoS One. 2012 Feb 8;7(2):e31129. doi: 10.1371/journal.pone.0031129 (PMC3275581; doi:10.1371/journal.pone.0031129)

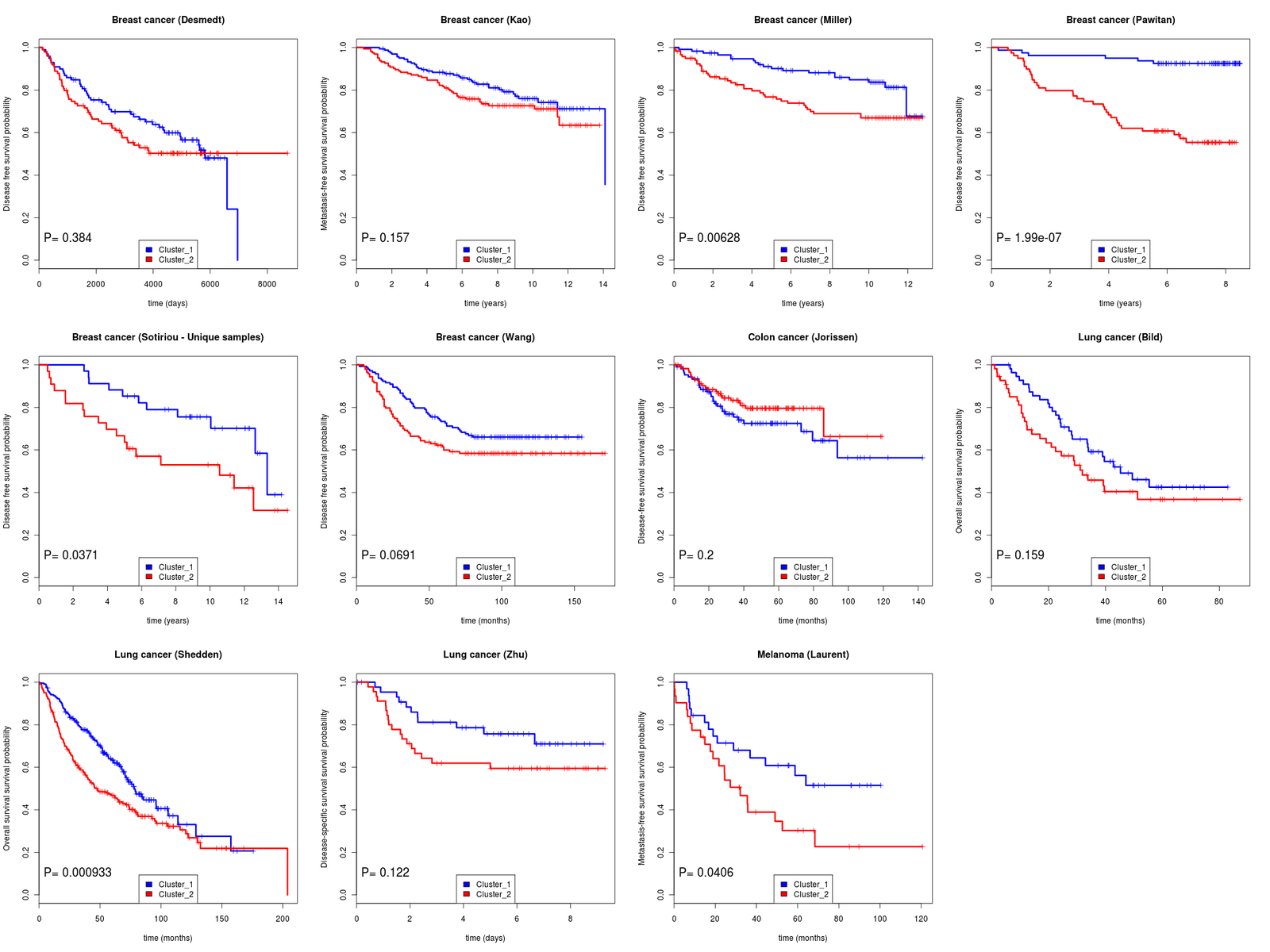

Supplement: Figure S1 — Performance of the signature derived from the Pawitan breast cancer dataset as a predictor of survival in other cancer datasets. P-values are from log-rank tests. (TIFF) [file pone.0031129.s001.tiff]

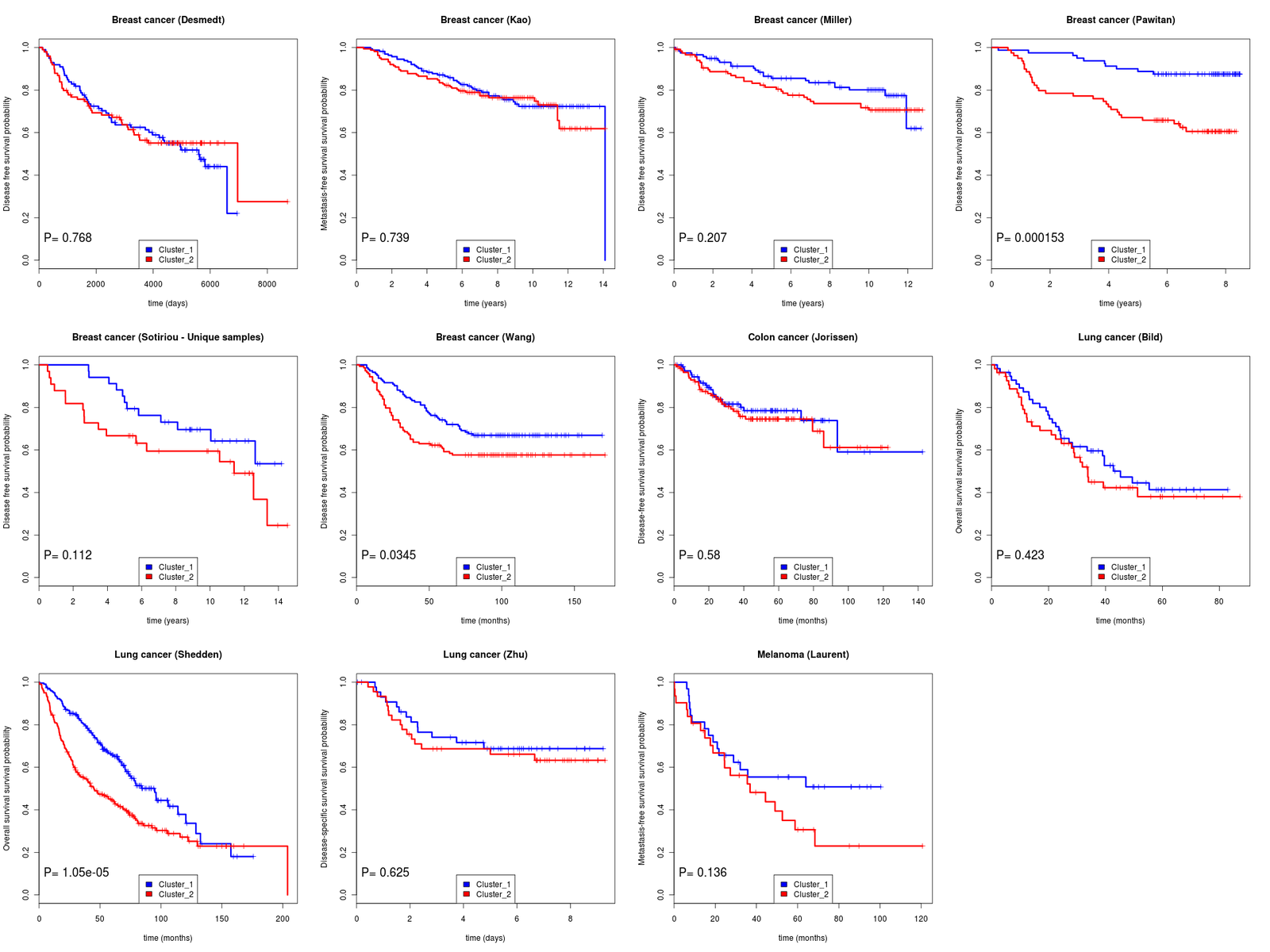

Supplement: Figure S2 — Performance of the signature derived from the Shedden lung cancer dataset as a predictor of survival in other cancer datasets. P-values are from log-rank tests. (TIFF) [file pone.0031129.s002.tif]
